# Remote Performance Evaluation of High-Precision Timekeeping Systems Based on BDS Real-Time PPP

**DOI:** 10.3390/s26185718

**Published:** 2026-09-09

**Authors:** Xiaonan Yang, Xiangbo Zhang, Jianfeng Wu, Ji Guo

**Affiliations:** 1 National Time Service Center, Chinese Academy of Sciences, Xi’an 710600, China; yangxiaonan@ntsc.ac.cn (X.Y.); wujianf@ntsc.ac.cn (J.W.); guoji@ntsc.ac.cn (J.G.); 2 College of Electronic, Electrical and Communication Engineering, University of Chinese Academy of Sciences, Beijing 100049, China; 3 Key Laboratory of Time Reference and Applications, Chinese Academy of Sciences, Xi’an 710600, China

**Keywords:** high-precision timekeeping system, remote performance evaluation, PPP-B2b, optical-fibre link, GNSS IPPP, frequency deviation, frequency stability

## Abstract

**Highlights:**

**What are the main findings?**

- A real-time remote monitoring and performance evaluation scheme based on BDS RTPPP is developed for high-precision timekeeping systems, in which receiver-clock constrained PPP processing is adopted for real-time station clock estimation. The developed system achieves frequency stability better than 8 × $10^{-15}$ at an averaging time of one day.
- Short-baseline and long-baseline BDS RTPPP performance evaluation experiments are carried out using optical-fibre and GNSS IPPP time transfer as references, respectively. Experimental results demonstrate that sub-nanosecond-level time-transfer agreement is better than 0.5 ns, enabling accurate performance evaluation of frequency deviation, frequency stability, and other metrics.

**What are the implications of the main findings?**

- BDS RTPPP time transfer technique provides a flexible remote real-time performance evaluation alternative for high-precision timekeeping systems, eliminating the limitations of traditional on-site accompanying and laboratory-submitted calibration.
- The presented methodology serves as a vital pathway to achieve real-time traceability and quantity unification of time and frequency across wide-area networks, with broad applicability across diverse research and industry fields.

**Abstract:**

Remote real-time performance evaluation of standard time and frequency is critical for full-scope time traceability and quantity unification. Conventional time and frequency calibration schemes predominantly adopt on-site accompanying calibration or laboratory submission, which are hampered by bulky equipment, high operational costs and poor flexibility, rendering them incompatible with continuous real-time monitoring and evaluation scenarios. While GNSS common-view and all-in-view techniques are available for remote performance evaluation, their limited precision cannot satisfy the demands of high-precision timekeeping systems with a frequency stability at the $10^{-15}$ level. This paper constructs a remote real-time performance evaluation system based on self-developed BDS real-time PPP (RTPPP) receivers. Two validation experiments are carried out: a 43 km short-baseline link between Lintong and Hangtian campus of the National Time Service Center (NTSC) referenced to an optical-fibre time transfer link, and a 1150 km long-baseline link between Lintong and Anhui referenced to a GNSS IPPP link. Experimental results indicate that the frequency stability of the BDS RTPPP link is better than 8 × $10^{-15}$ at an averaging time of one day. For the single hydrogen-maser timekeeping system located at Hangtian, the average frequency deviation and frequency stability measured via BDS RTPPP reach 1.93 × $10^{-15}$ and 2.07 × $10^{-15}$/1 d, respectively, showing close agreement with the independently calibrated optical-fibre reference. For the Anhui timekeeping system, the one-day average frequency deviation is 8.90 × $10^{-15}$ and frequency stability reaches 7.95 × $10^{-15}$ after one day of averaging, which are in close agreement with the GNSS IPPP reference results. The overall findings verify that BDS RTPPP based on PPP-B2b service is qualified for remote real-time performance evaluation and monitoring of high-precision timekeeping systems.

## 1. Introduction

Conventional time and frequency calibration schemes are tailored for discrete time and frequency devices and predominantly adopt on-site testing, equipment relocation for laboratory submission. While these techniques can deliver decent accuracy, they are not readily applicable to the remote real-time performance evaluation of integrated timekeeping systems assembled with multiple hydrogen masers and cesium clocks. GNSS Common-View(CV) and All-in-View(AV) techniques offer an alternative for remotely evaluating standard time and frequency references and timekeeping systems. Nevertheless, such methods mostly rely on post-processing workflows, and their limited precision imposes notable constraints on practical performance [1,2,3]. Furthermore, GNSS CV/AV struggles to characterize the long-term frequency stability of high-precision timekeeping systems with active hydrogen masers commonly deployed in national timing laboratories, which attain daily frequency stability at the level of $10^{-15}$. By comparison, GNSS time transfer with carrier-phase measurements features high precision, global coverage, flexible deployment, and moderate cost [4,5]. It has evolved into a core technique route for the remote performance verification and performance assessment of timekeeping systems [6].

As a representative GNSS carrier phase time transfer technique, Precise Point Positioning (PPP) has been adopted within the time and frequency community for over 16 years [7,8,9]. The classic PPP model features a concise framework, fewer unknown parameters and low computational complexity. Nevertheless, it yields float ambiguities owing to the assimilation of code-pseudorange noise during parameters estimation. Numerous studies have demonstrated that float PPP delivers sub-nanosecond time transfer accuracy, with frequency stability exceeding 1 × $10^{-15}$ at averaging times of one day [10,11,12]. By recovering the integer characteristics of carrier-phase ambiguities, IPPP enables ambiguity-fixed PPP solutions and improves the precision and continuity of GNSS time transfer. Research confirms that IPPP tightens the constraint imposed by carrier-phase observations on station clock estimation, thereby elevating time transfer accuracy relative to float PPP. In addition, IPPP further improves the time transfer continuity as well as long-term frequency stability [13,14,15,16]. Even so, both float PPP and IPPP mostly depend on post-processed precise orbit, clock, and bias products, which severely limits their real-time performance. They are thus only applicable as high-precision post-processing reference links and cannot independently satisfy the real-time performance evaluation demands of timekeeping systems.

To satisfy the real-time performance evaluation demands of high-precision timekeeping systems, particularly the hydrogen-cesium combined systems with active hydrogen maser serving as the primary maser clock, GNSS real-time PPP(RTPPP) time transfer is adopted as the technical solution. For IGS-supported RTPPP, the receiver decodes State Space Representation (SSR) corrections for satellite orbit, clock, and code bias in real time, and derives precise satellite orbit and clock products from broadcast ephemerides [17,18,19]. Conventional RTPPP retrieves IGS SSR corrections via the Networked Transport of RTCM via Internet Protocol (NTRIP). Although SSR corrections can be readily accessed via the network transmission, their continuity breaks down under network outages. Such interruptions may trigger PPP solution discontinuity or necessitate reconvergence. Satellite clock products are fundamental to maintaining the high-precision performance of RTPPP time transfer. Accordingly, uninterrupted real-time clock corrections effectively boost the reliability of PPP time transfer [20].

The BDS-3 PPP-B2b service offers an innovative way to broadcast real-time corrections for PPP time transfer. It delivers real-time orbit, clock, and code bias corrections directly via BDS-3 GEO satellites. After receiving navigation messages with GNSS receivers, users can calibrate the orbit, clock, and code bias within broadcast ephemerides in real time for PPP station clock estimation [21,22,23,24]. Advancements in the signal structure, precise orbit determination capability and onboard atomic clock performance of BDS-3 satellites have laid a solid foundation for high-precision positioning, navigation, and timing (PNT) as well as PPP applications [25,26]. Unlike IGS SSR-based RTPPP, which relies on internet-dependent correction streams, BDS PPP-B2b features superior autonomy and easy deployment and fits regional real-time operational scenarios, delivering an alternative technique route for real-time comparison. Existing studies have demonstrated that the PPP-B2b service mitigates orbit and clock errors in broadcast ephemerides, delivers sub-nanosecond real-time station clock estimation accuracy, and enables RTPPP time transfer across China and adjacent regions. The rollout of satellite-borne high-precision services such as Galileo HAS further demonstrates that real-time precise corrections are evolving from ground-network-dependent data links toward direct satellite broadcasting [27,28]. This trend provides crucial support for high-precision RTPPP time transfer.

This work aims to analyze and verify the remote real-time performance evaluation capability of high-precision timekeeping systems via BDS RTPPP time transfer based on the PPP-B2b service. In this study, “remote real-time performance evaluation” refers to the continuous real-time acquisition of time and frequency comparison results through the BDS RTPPP link, whereas the quantitative performance assessment is performed using the accumulated comparison data. In Section 2, the fundamental characteristics of classical float-ambiguity PPP and IPPP with integer ambiguity resolution are introduced. Furthermore, a receiver-clock constrained parameter estimation strategy is adopted in the BDS RTPPP processing, in which a second-order polynomial receiver clock model is incorporated into the Kalman filter framework. Receiver-clock constrained PPP has previously been shown to improve the continuity and robustness of PPP-B2b station-clock estimation [23]. In the present study, this strategy is employed as part of the RTPPP processing chain, while the main focus is placed on establishing and experimentally validating a remote real-time performance evaluation framework for high-precision timekeeping systems.

## 2. Methods and Principles

### 2.1. Float-Ambiguity Classical PPP Time Transfer

Float-ambiguity classical PPP relies on GNSS dual-frequency ionosphere-free combined observations of code-pseudorange and carrier phase. Precise orbit, clock, and bias products released by IGS or corresponding Analysis Centers (ACs) are utilized to remove satellite-related systematic errors. After linearizing the observation equations, sequential least squares or the Extended Kalman Filter (EKF) is employed to solve for unknown parameters, namely station coordinates, clock, zenith wet tropospheric delay and carrier-phase ambiguity. Once the PPP-derived station clocks at both ends of the baseline are obtained, epoch-wise differencing station clock over the same time span yields the time transfer solution.

With the rapid development of Global Navigation Satellite Systems (GNSS) and MGEX products provided by IGS analysis centers, combined multi-GNSS PPP time transfer can simultaneously utilize all constellation observations from GPS, BDS, Galileo, and other systems. Compared with single PPP time transfer link, multi-GNSS PPP not only improves the robustness of time transfer links, but also improves the frequency stability of time transfer results. Especially, with the release of multi-GNSS bias products by the IGS Analysis Center Coordinator (ACC), multi-GNSS PPP with integer ambiguity resolution can be achieved. Relevant studies have shown that combined multi-GNSS PPP with ambiguity resolution can further improve PPP performance, making it more suitable for high-precision time and frequency transfer applications [29,30]. Nevertheless, inter-system biases (ISBs) should be rigorously addressed, especially for BDS combined PPP time transfer [31]. In time-frequency applications, the estimated receiver clock offset rather than positioning accuracy is the primary parameter of interest.

### 2.2. IPPP Time Transfer with Integer Phase Clock Ambiguity Resolution

It is widely recognized that classical float-ambiguity PPP suffers from the day-boundary discontinuities caused by the code measurement noise, which limits its capability for continuous precise time transfer and weakens long-term frequency stability. To address this issue, integer ambiguity fixed PPP methods have been developed, among which the integer phase clock strategy provided by the Centre National d’Études Spatiales(CNES) has become an important technical basis for continuous IPPP time and frequency transfer [14,32,33]. The CNES integer phase clock scheme absorbs satellite fractional phase deviations into satellite clock products, thus separating integer components of carrier phase ambiguity and enabling ambiguity resolution in PPP. To further improve ambiguity-fixing performance, the IPPP model integrates inter-satellite single-difference constraints and observable-specific signal bias (OSB) products released by CODE for enhanced bias correction [34]. OSB products deliver frequency-dependent code and phase corrections for individual GNSS signals, which eliminate fractional phase deviation from ambiguity parameters and improve the reliability of undifferenced integer ambiguity resolution [34]. Combined with integer phase clock constraints, OSB-calibrated ambiguities can converge to stable integer values.

This study employs undifferenced pseudorange and carrier-phase observation models to implement PPP time and frequency transfer. Before parameter estimation, dominant systematic errors including orbital deviations, tidal displacements, antenna phase center variations and phase wind-up effects are corrected following IGS and IERS standard conventions [35]. After compensating for these systematic errors and conducting joint calibration using CNES integer phase clocks alongside CODE OSB products, fractional phase biases contaminating ambiguities are subsequently removed, laying the foundation for robust integer ambiguity fixing. Inter-satellite single-difference processing further eliminates receiver clock offsets and hardware-dependent biases, leaving only differenced ambiguities with strict integer characteristics. The wide-lane ambiguity candidates are first determined using the Melbourne–Wübbena combination, followed by narrow-lane ambiguity validation based on the LAMBDA estimator [36]. Once all ambiguities are constrained to integer values, station clocks are re-estimated to generate continuous, low-noise and high-stability time sequences. The joint correction scheme combining integer phase clocks and OSB bias products enables IPPP to effectively mitigate the day-boundary discontinuities and long-term temporal drift. It delivers superior short-term time precision and long-term frequency stability compared with conventional float PPP.

### 2.3. Parameter Estimation Algorithm for BDS Real-Time PPP with Receiver-Clock Constrained

**Figure 1:**
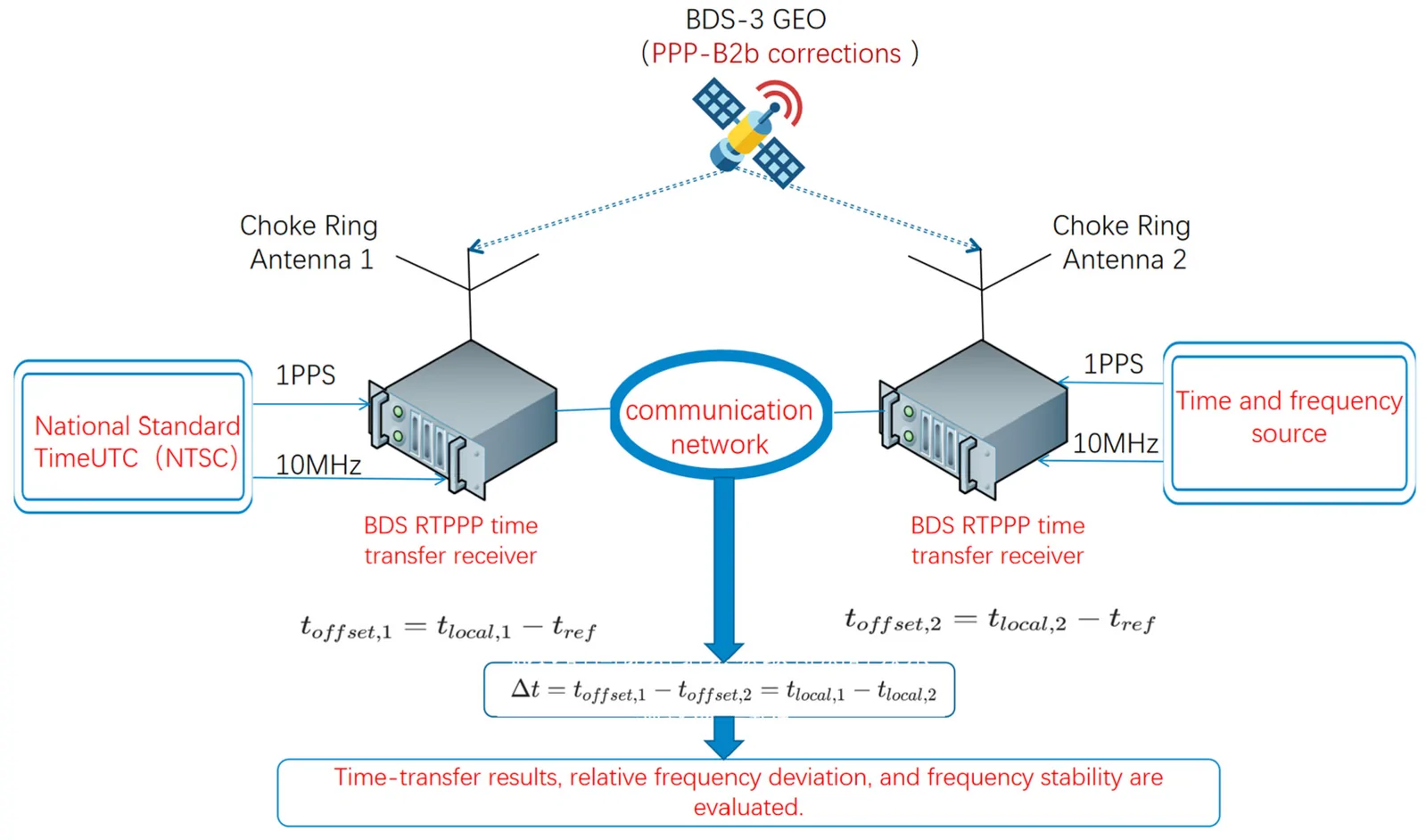
Schematic diagram of the BDS RTPPP time transfer based on PPP-B2b service.

The fundamental principle of BDS RTPPP time transfer based on PPP-B2b service is illustrated in Figure 1. This technique leverages real-time precise corrections disseminated via BDS-3 GEO satellites under the PPP-B2b service to correct the satellite orbit, station clock, and Differential Code Biases (DCBs) embedded within the broadcast ephemerides, yielding high-precision orbit and clock products that satisfy the requirements for PPP data processing [21,22].

The PPP-B2b-corrected satellite orbit and clock products support real-time estimation of PPP station clock. At a single observation station, the receiver adopts the 10 MHz frequency signal and 1 PPS pulse output from the local timekeeping system as external time and frequency references. The PPP station clock is estimated based on code-pseudorange and carrier-phase observations, which quantify the time offset of the local timekeeping system relative to the BeiDou Time (BDT) scale. By independently estimating station clocks at two stations, the time offset between the two timekeeping systems can be acquired by epoch-wise differencing of the estimated PPP station clocks.

In conventional BDS real-time PPP, the receiver station clock is generally treated as epoch-wise uncorrelated white noise. This strategy neglects the continuous and slowly varying characteristics of hydrogen and cesium atomic clocks equipped in high-precision timekeeping receivers. When SSR corrections experience short-term interruptions or satellite observation geometry deteriorates, strong coupling may occur between station coordinates and receiver clock parameters, leading to filter reconvergence and degradation of time transfer continuity. To improve the continuity and robustness of receiver clock estimation, the second-order polynomial dynamic model is employed in this study to describe the temporal evolution of the receiver station clock, and the corresponding temporal constraint is incorporated into the Kalman filter framework for real-time PPP parameter estimation.

Dual-frequency ionosphere-free (IF) combinations are adopted to eliminate the first-order ionospheric delay. The observation equations can be expressed as:

(1) $$P_{IF}=\rho +c(dt_{r}-dt^{s})+T_{z}+\varepsilon_{P}$$

(2) $$\Phi_{IF}=\rho +c(dt_{r}-dt^{s})+T_{z}-\lambda_{IF}N_{IF}+\varepsilon_{\Phi }$$

where $P_{IF}$ and $\Phi_{IF}$ denote the ionosphere-free combined pseudorange and carrier-phase observations, respectively; ρ represents the instantaneous geometric distance between the satellite and receiver; c is the speed of light in vacuum; $dt_{r}$ and $dt^{s}$ represent the receiver and satellite station clock, respectively, where $dt^{s}$ is corrected using BDS PPP-B2b SSR products; $T_{z}$ is the zenith tropospheric delay; $\lambda_{IF}$ and $N_{IF}$ denote the wavelength and ambiguity of the ionosphere-free combination; and $\varepsilon_{P}$ and $\varepsilon_{\Phi }$ represent the measurement noise of pseudorange and carrier-phase observations.

The receiver station clock is modelled using a second-order polynomial model:

(3) $$dt_{r}(t)=a_{0}+a_{1}(t-t_{0})+\frac{1}{2}a_{2}{\left(t-t_{0}\right)}^{2}$$

where $t_{0}$ represents the reference epoch; $a_{0}$, $a_{1}$, and $a_{2}$ correspond to the clock bias, frequency offset, and frequency drift, respectively.

Instead of estimating $dt_{r}$ independently at each epoch, the polynomial coefficients are introduced as unknown parameters, and an extended state vector is constructed. The state vectors of the conventional epoch-wise clock estimation strategy and the receiver-clock constrained strategy adopted in this study are defined as:

(4) $$X_{classic}={\left[x,y,z,dt_{r},T_{z},N_{IF}^{1},\cdots N_{IF}^{n}\right]}^{T}$$

(5) $$X={\left[x,y,z,a_{0},a_{1},a_{2},T_{z},N_{IF}^{1},\cdots ,N_{IF}^{n}\right]}^{T}$$

where x, y, and z denote the three-dimensional receiver coordinates.

Considering the filter interval Δt, the discrete state transition model of the clock parameters is expressed as:

(6) $${\left[\begin{array}{c} a_{0}\\ a_{1}\\ a_{2} \end{array}\right]}_{k+1}=\left[\begin{array}{ccc} 1 & \Delta t & \frac{1}{2}\Delta t^{2}\\ 0 & 1 & \Delta t\\ 0 & 0 & 1 \end{array}\right]{\left[\begin{array}{c} a_{0}\\ a_{1}\\ a_{2} \end{array}\right]}_{k}+\omega_{a}$$

The complete state transition equation can therefore be written as:

(7) $$X_{k+1}=\Phi X_{k}+\omega_{k}$$

where Φ represents the state transition matrix and $\omega_{k}$ denotes the process noise vector.

Previous studies have demonstrated the effectiveness of receiver-clock constrained PPP for time transfer based on BDS-3 PPP-B2b service and compared it with the conventional white-noise receiver clock strategy [23]. Those works revealed that receiver-clock constrained modelling is capable of suppressing abnormal station clock variations in PPP, mitigating solution reconvergence triggered by data anomalies, and substantially improving short-term frequency stability. Nevertheless, the receiver-clock constrained PPP itself is not regarded as the core methodological contribution of the present study. Instead, this work adopts receiver-clock constrained BDS RTPPP as the real-time performance evaluation strategy, with emphasis placed on the full remote performance evaluation link, including common-clock link delay calibration, independent short and long baseline reference comparisons, as well as quantitative assessment for the agreement and metrological calibration uncertainty.

**Figure 2:**
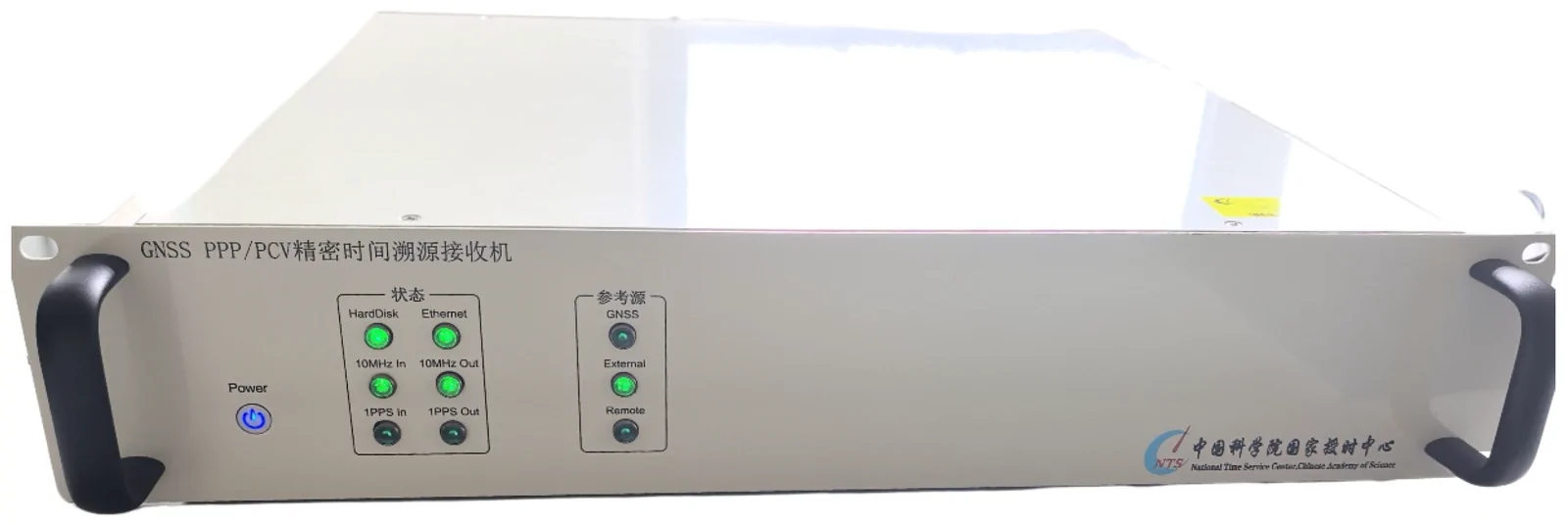
Photograph of the BDS PPP-B2b real-time PPP receiver.

**Figure 3:**
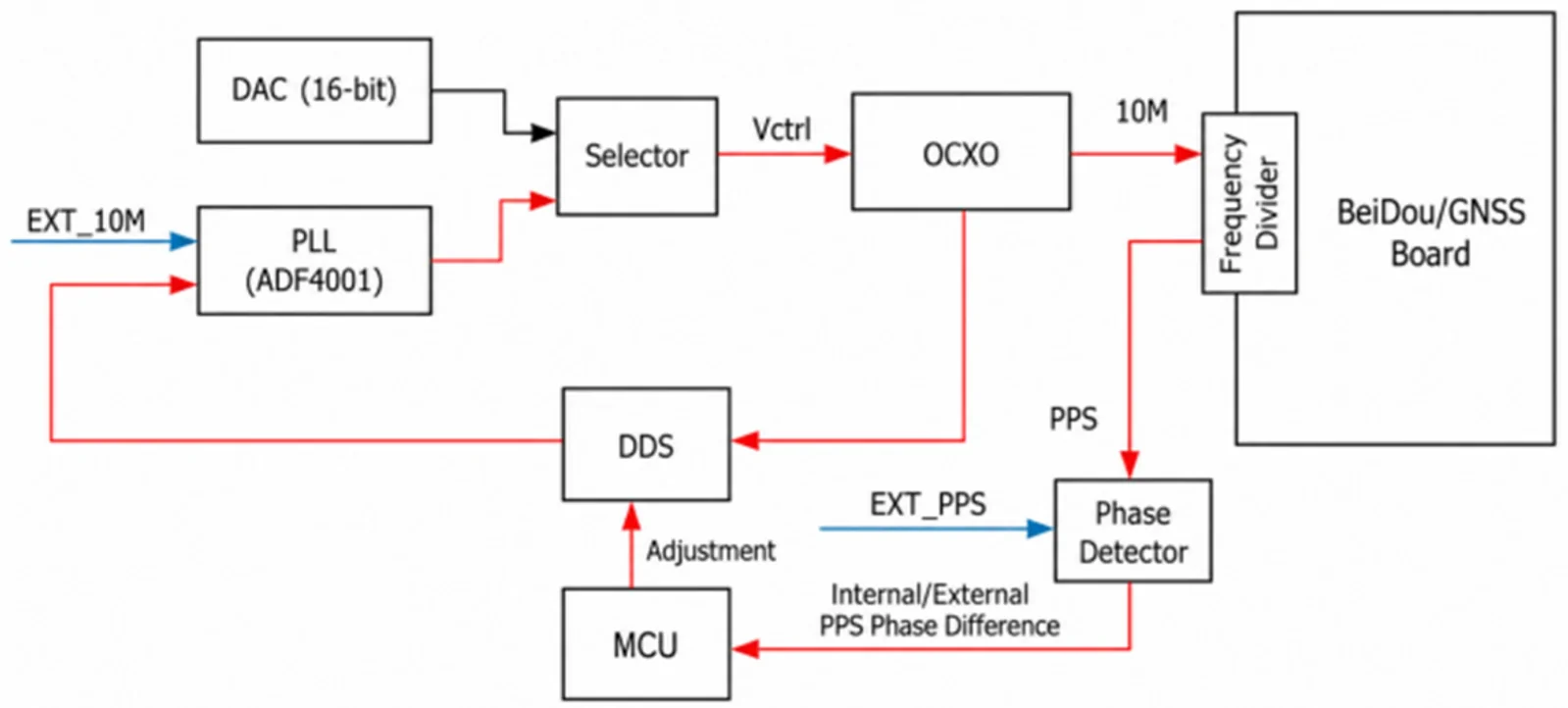
Schematic diagram of time synchronization for BDS RTPPP receiver.

To construct such an evaluation link, a remote real-time performance evaluation system is developed using a self-developed BDS RTPPP time and frequency transfer receiver. The physical prototype of the receiver and its internal synchronization scheme are presented in Figure 2 and Figure 3, respectively. A phase-locked loop (PLL) is employed to lock the output frequency of the internal Oven-Controlled Crystal Oscillator (OCXO) to the external reference frequency. Benefiting from this synchronous architecture, RTPPP solutions can precisely characterize the time comparisons between two remote stations.

### 2.4. Evaluation Metrics for Remote Performance Evaluation of Timekeeping Systems

For high-precision timekeeping systems, relative frequency deviation and frequency stability are adopted as the primary performance indicators in this study. The relative frequency deviation characterizes the average frequency offset of the timekeeping system under test with respect to the reference over a specified time interval and is calculated as follows:

(8) $$y_{AB}(\tau )=\frac{x_{M+1}-x_{1}}{\tau }$$

where $y_{AB}(\tau )$ denotes the relative frequency deviation of the timekeeping system under test with respect to the reference over the interval τ; $x_{1}$ and $x_{M+1}$ denote the time transfer results at the first epoch of the current batch and the first epoch of the subsequent batch, respectively. When one day is taken as one batch and the sampling interval is 30 s, M = 2880.

The Modified Allan Deviation (MDEV) is employed to characterize the frequency stability of the time transfer results and to distinguish different types of phase and frequency noise. It is calculated as follows:

(9) $$Mod\sigma_{y}(\tau )=\sqrt{\frac{1}{2m^{2}\tau^{2}(N-3m+1)}\sum_{j=1}^{N-3m+1}{\left\{\sum_{i=j}^{j+m-1}\left[x_{i+2m}-2x_{i+m}+x_{i}\right]\right\}}^{2}}$$

Time Deviation (TDEV), derived from MDEV, is further employed to characterize the time stability of the time-transfer results. In this study, MDEV and TDEV are used as statistical stability metrics for the comparison results.

For comparison with an independent reference link, the mean difference is used to characterize systematic agreement, while the STD, TDEV, and MDEV of the difference series describe statistical dispersion, time stability, and frequency stability, respectively. These stability metrics are used to characterize the statistical uncertainty contribution of the BDS RTPPP comparison results. In contrast, the Type B systematic uncertainty contribution of BDS RTPPP cannot be determined from the present comparison data alone, because it also depends on the metrological calibration uncertainty of the reference link and other systematic contributions. Thus, the absolute metrological calibration uncertainty of BDS RTPPP cannot be inferred solely from these metrics.

Furthermore, the frequency stability and relative frequency deviation performance of the selected reference link should be sufficiently superior to those of the timekeeping system under test to ensure reliable comparative evaluation.

## 3. Experimental Design and Data Processing

To verify the feasibility of remote real-time performance evaluation for high-precision timekeeping systems via B2b-correction-based BDS RTPPP, three stations were selected: the Lintong campus of the National Time Service Center, the Hangtian campus, and a station located in Anhui (Here, T denotes the local atomic timescale (TA), e.g., T(Hangtian) and T(Anhui)). Lintong hosts UTC(NTSC), which contributes to TAI/UTC computation with a daily averaged frequency stability better than 1 × $10^{-15}$; Hangtian operates a passive-hydrogen-maser-based timekeeping system reproducing UTC(NTSC), with a daily frequency stability of approximately 2.5 × $10^{-15}$; the Anhui station deploys multiple hydrogen masers and cesium clocks, achieving a frequency stability below $10^{-15}$ after one-day averaging.

BDS RTPPP remote real-time performance evaluation devices were employed at above three stations, taking 1 PPS and 10 MHz signals from the respective local timekeeping systems as external time-frequency references. A short-baseline link of approximately 43 km between Lintong and Hangtian and a long-baseline link of over 1150 km between Lintong and Anhui were established. For the short-baseline comparison, a self developed two-way optical-fibre time and frequency transfer system was adopted as the independent reference. The instrument delay was determined via zero baseline Common Clock Difference (CCD) experiments, giving a standard uncertainty of 8.2 ps. Delays of the optical-fibre link were calibrated using a round trip loop-back strategy, with a calibration uncertainty of approximately 19.6 ps. Since no optical-fibre link is available between Lintong and Anhui, a GNSS IPPP time-transfer link built upon Septentrio PolaRx5TR receivers(Septentrio Satellite Navigation, Leuven, Belgium) was constructed to serve as the independent reference for the long-baseline experiment.

Equipment deployment at the three stations is summarized in Table 1. Relative frequency deviation and frequency stability are finally computed to quantitatively assess the overall frequency performance.

The observation period of the Lintong–Hangtian short baseline spans seven days, covering the Modified Julian Date (MJD) 61167 to MJD 61173. However, no valid observations are available for the optical-fibre link on MJD 61167. Accordingly, the BDS RTPPP and optical-fibre time transfer results for the short-baseline only cover six days from MJD 61168 to MJD 61173. The long-baseline campaign between Lintong and Anhui also lasts eight days, from MJD 61150 to MJD 61157. For the eight-day IPPP data processing, the length of a batch is one day. After cycle slip detection and repair were performed on the GNSS observations collected by the receivers, the IPPP parameter estimation scheme and error correction strategies were adopted as shown in Table 2.

Before the RTPPP and IPPP remote comparison tests, zero-baseline common-clock differencing is utilized to calibrate the link delay for both schemes. The calibrated link delays were subsequently applied to the short and long-baseline experiments to compensate for the corresponding fixed instrumental and link delay contributions before the comparative analysis.

**Figure 4:**
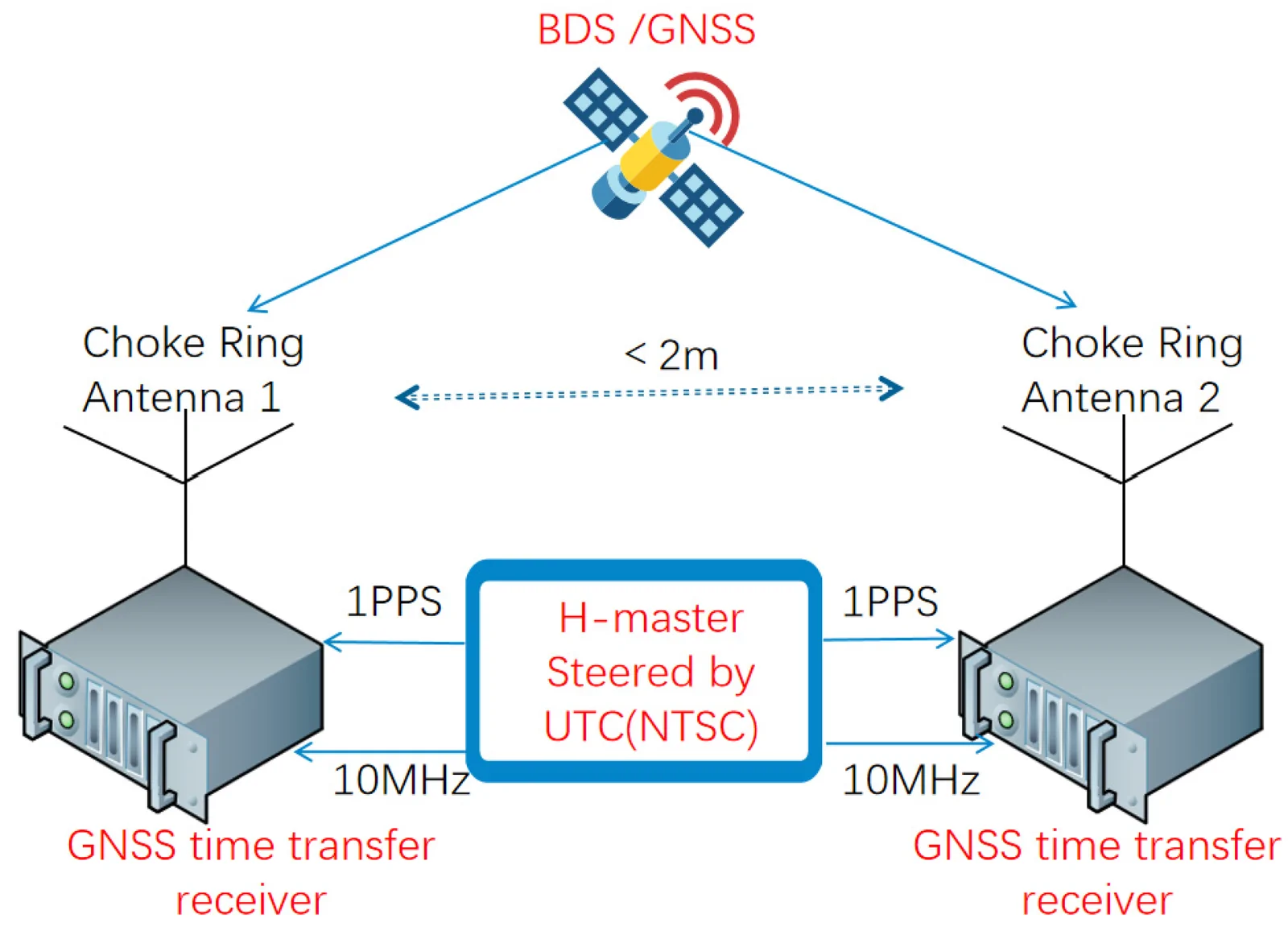
Schematic diagram of the zero-baseline CCD experiment.

The zero-baseline experiment was conducted at the Lintong campus of NTSC. The reference and user terminals were placed within 2 m of each other and were both referenced to the 1 PPS and 10 MHz signals generated by UTC(NTSC). The experimental configuration is illustrated in Figure 4, and the observation period covered MJD 60894–60898.

Since the RTPPP and the IPPP links adopt the same external references, their time comparisons mainly reflect fixed hardware delays from antennas, cables, 1 PPS channels. The fluctuations of their mutual difference characterize the random error magnitude of the two links.

**Figure 5:**
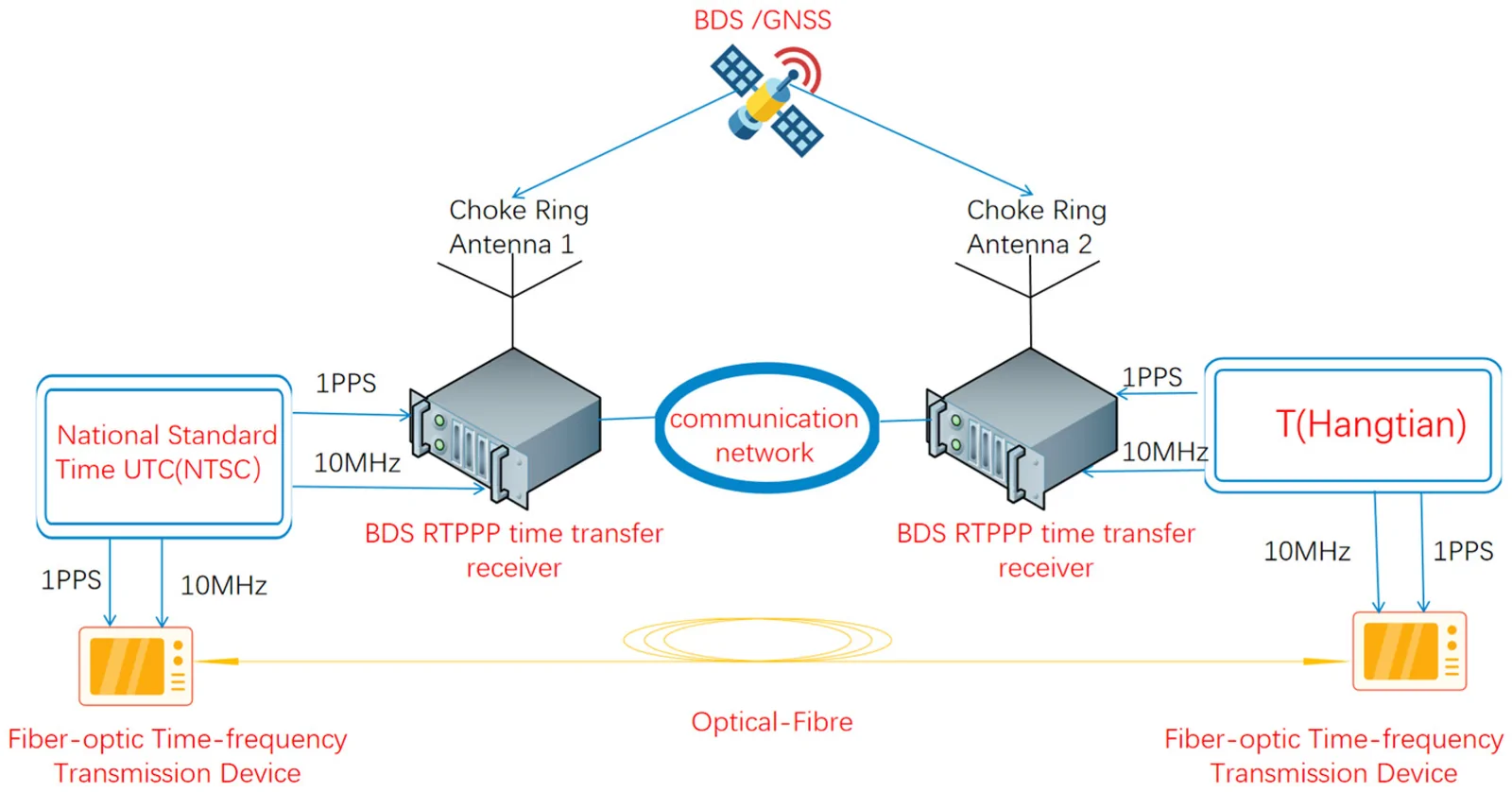
Scheme of Short-Baseline Comparison and Optical-Fibre Performance Evaluation for the Lintong-Hangtian link.

After calibrating link delays, a single hydrogen maser timekeeping system at Hangtian campus was taken as the device under test to verify the remote real-time performance evaluation capability of BDS RTPPP. The 43 km optical-fibre time comparison link between Lintong and the Hangtian campus was utilized as an external reference for evaluating RTPPP results. The experimental principle is shown in Figure 5.

**Figure 6:**
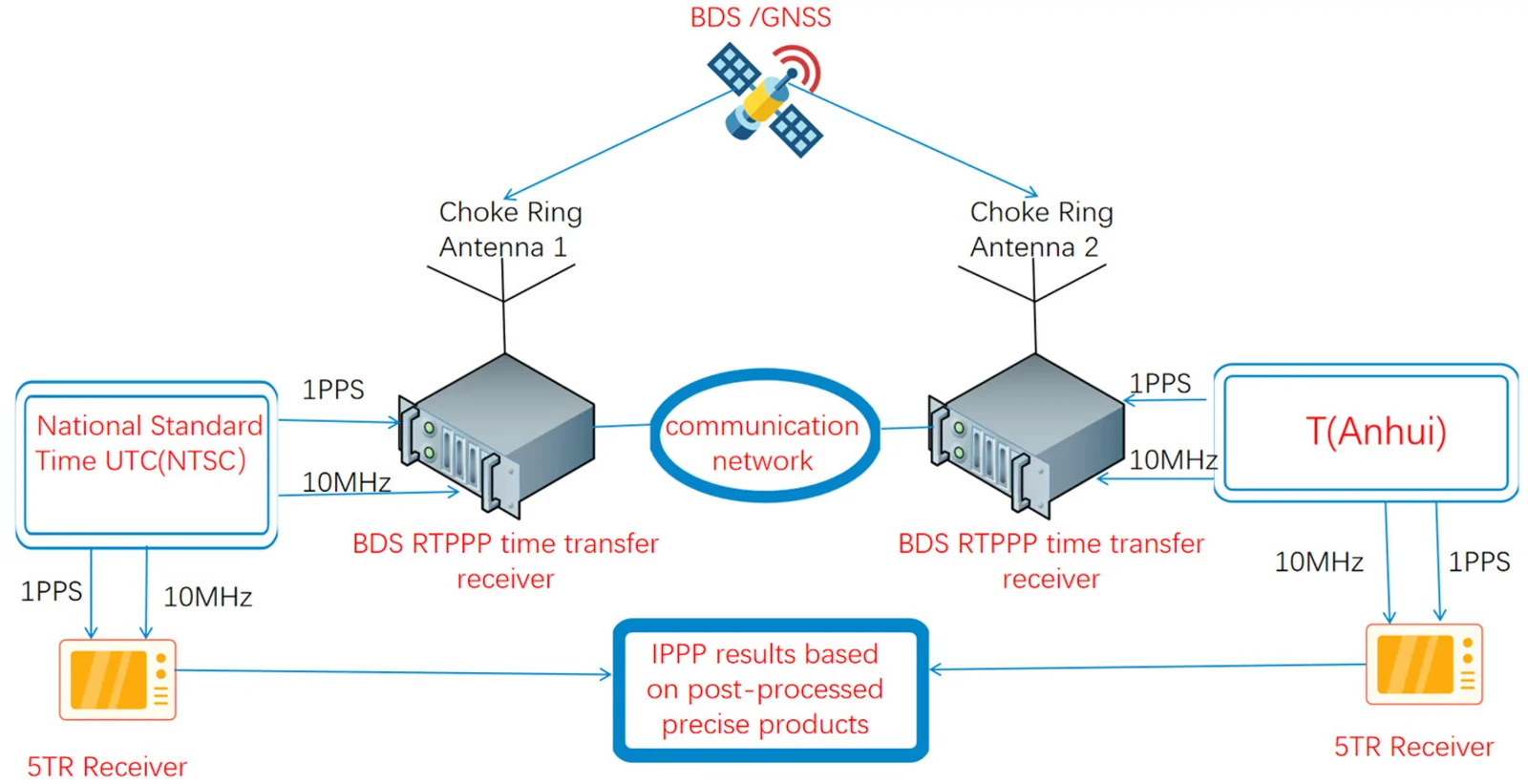
Scheme of Long-Baseline Comparison and IPPP Evaluation for the Lintong-Anhui link.

We verify the long-distance evaluation capability of the BDS RTPPP remote real-time performance evaluation system with the timekeeping system located in Anhui. Its hydrogen maser is steered to generate the local time scale T(Anhui), whose 1PPS and 10 MHz outputs provide external references for the BDS RTPPP system to build the over 1150 km Lintong-Anhui link, the multi-GNSS IPPP time comparison link acts as the external reference for external conformity assessment of RTPPP evaluation results, with the experimental principle given in Figure 6.

For the long-baseline reference, the IPPP processing strategy described in Section 2.2 was employed. The detailed parameter estimation and correction settings are summarized in Table 2.

## 4. Experimental Results and Analysis

### 4.1. Zero-Baseline Common Clock Difference Experiments

**Figure 7:**
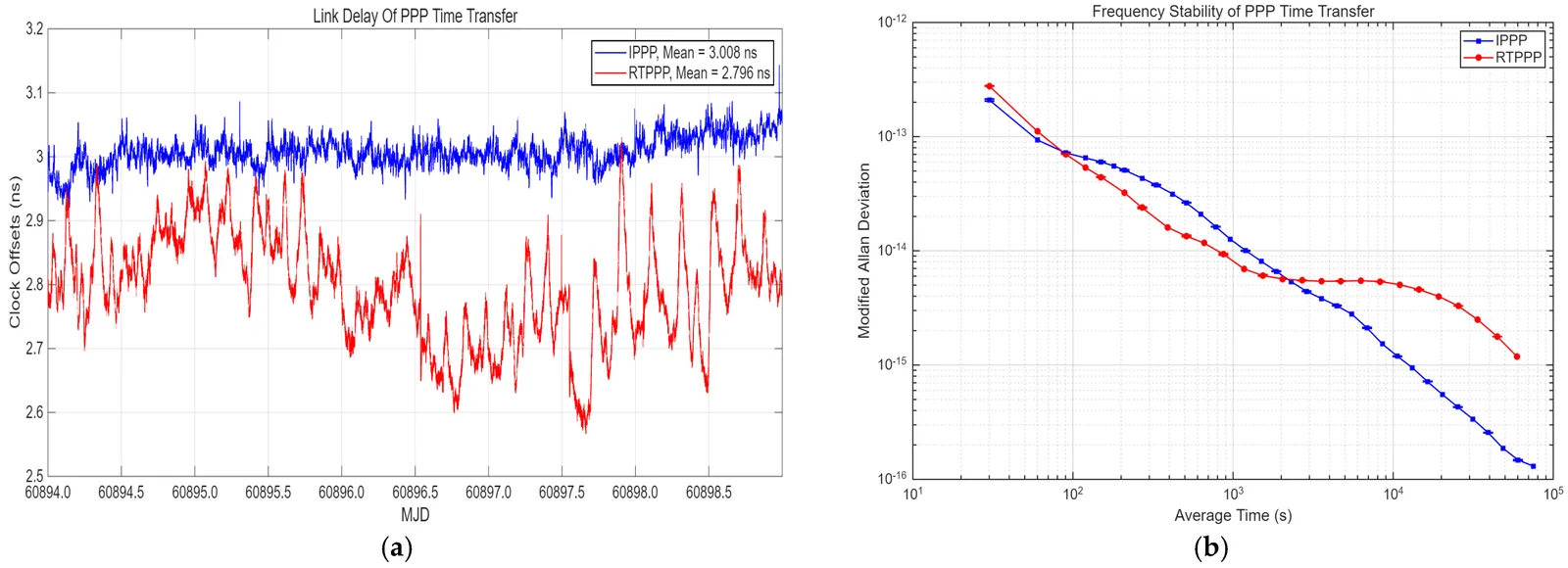
(**a**) Common-Clock Difference Results of the BDS RTPPP and IPPP Links; (**b**) Frequency Stability of the Common-Clock Comparison Results of the Two Links.

Figure 7 presents the zero-baseline CCD results and the corresponding MDEV results for the BDS RTPPP and IPPP links. Under the common-clock configuration described in Section 3, the observed fluctuations mainly reflect the internal statistical variation in the two-comparison links.

As illustrated in Figure 7a, the CCD results of both the BDS RTPPP and IPPP links remain fairly stable without abrupt jumps. The mean link delays of the two links are 2.796 ns and 3.008 ns, respectively. Their corresponding standard deviations (STD) of CCD solutions are 0.077 ns and 0.017 ns accordingly. Compared with the BDS RTPPP link, the IPPP link delivers notably suppressed random noise, demonstrating lower short-term random fluctuations. While the CCD results of the RTPPP link fluctuate within a range of 0.5 ns and presents larger variations relative to the IPPP counterpart, and the fluctuations remain at the sub-nanosecond level. The quasi-periodic peaks are likely associated with changes in satellite geometry and the quality/availability of real-time PPP-B2b corrections. Because no corresponding discontinuity is observed in the IPPP solution using post-processed precise products, the peaks are more likely related to the real-time correction and filtering process than to the common reference clock. A definitive attribution would require additional satellite-geometry and correction-age diagnostics, which were not recorded in the present experiment.

As presented in Figure 7b, the BDS RTPPP link achieves superior frequency stability relative to the IPPP link for averaging times shorter than 1000 s. By contrast, the IPPP link outperforms RTPPP in frequency stability when the averaging time exceeds 1000 s. This indicates that BDS RTPPP delivers better short-term stability, while IPPP is advantageous in long-term frequency stability. The discrepancy primarily arises from the higher quality of post-processed precise orbits and clock products compared with broadcast orbit/clock parameters corrected by real-time B2b correction streams. Additionally, IPPP enables more comprehensive mitigation of random errors. In contrast, BDS RTPPP is susceptible to large random errors originating from decoded B2b corrections as well as broadcast orbit and clock products. Despite its higher link resolution, RTPPP exhibits inferior frequency stability relative to IPPP.

The frequency stability after averaging time of one day for the two links were not calculated due to the limited duration of the time comparison campaign and insufficient data volume. Nevertheless, the MDEV curves show that the frequency stability of both the BDS RTPPP and IPPP links reaches the $10^{-15}$ level at long averaging times. These results indicate that the BDS RTPPP link exhibits good frequency stability under the zero-baseline common-clock configuration. It should be emphasized that the MDEV results characterize the statistical frequency stability of the links and are not used here to quantify systematic metrological calibration errors or absolute metrological calibration uncertainty.

### 4.2. Short-Baseline Comparison Experiments

**Figure 8:**
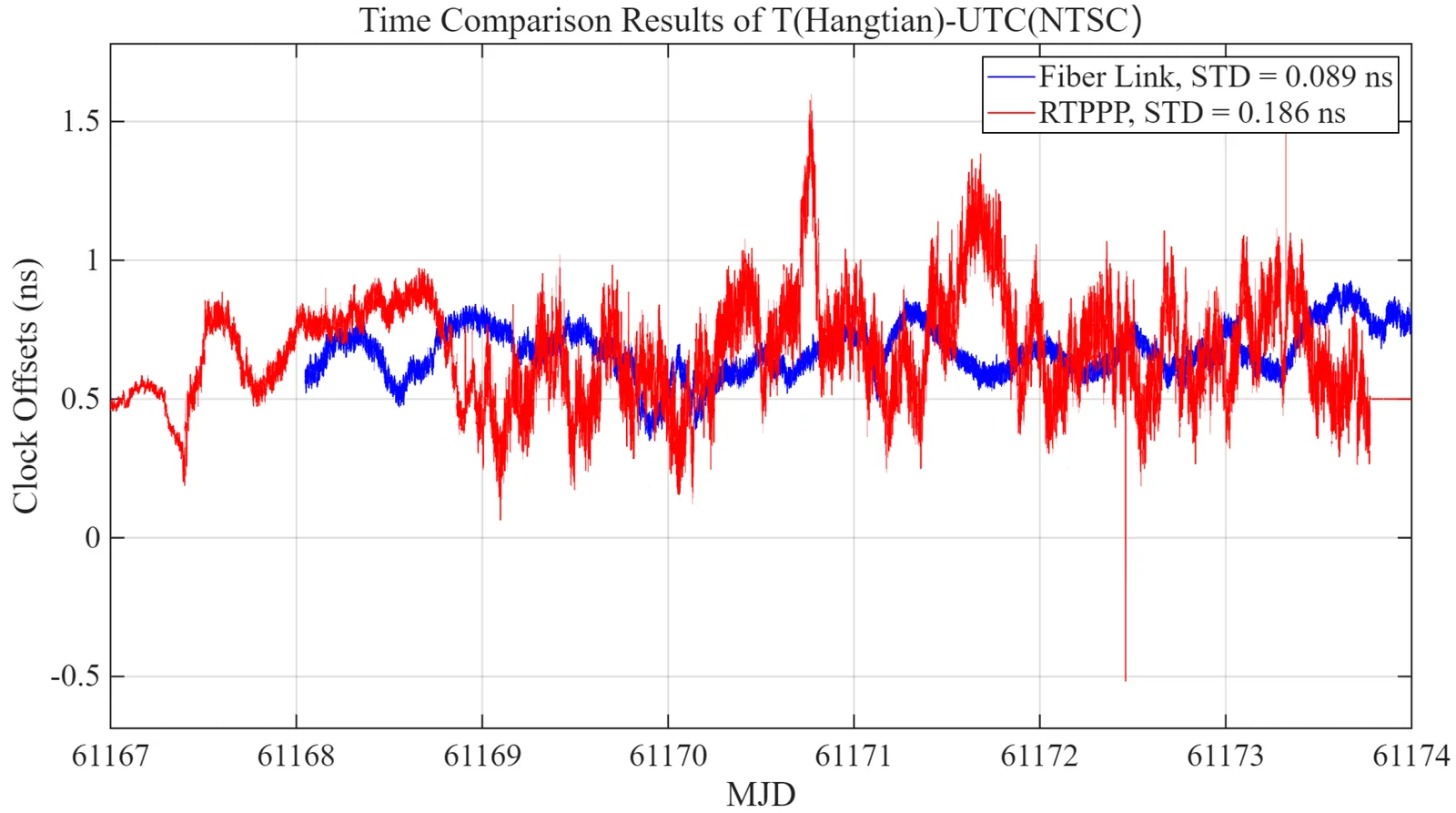
T(Hangtian) − UTC(NTSC) derived from the BDS RTPPP and optical-fibre links.

Using the short-baseline configuration described in Section 3, the BDS RTPPP time comparison results for T(Hangtian) were compared with those obtained from the independently calibrated optical-fibre reference link. After link-delay compensation, the T(Hangtian) − UTC(NTSC) time-offset results obtained from the two links are shown in Figure 8.

Figure 8 presents the time comparison results obtained from the BDS RTPPP and optical-fibre links, where the blue curve represents the optical-fibre results and the red curve represents the BDS RTPPP results. Both links exhibit broadly consistent temporal variations, while the optical-fibre results show lower short-term fluctuations. The STDs of the individual time offset series are 0.186 ns for BDS RTPPP and 0.0899 ns for the optical-fibre link, respectively. These values characterize the statistical fluctuations of the individual time comparison results rather than the metrological calibration accuracy of the BDS RTPPP link.

To directly evaluate the agreement between the two independent links, the BDS RTPPP and optical-fibre results were aligned over their common observation interval from MJD 61168 to MJD 61173, excluding MJD 61167 because no valid optical-fibre observations were available. Following the evaluation criteria defined in Section 2.4, the absolute mean difference between the BDS RTPPP and optical-fibre results is 0.064 ns, indicating close agreement between the two independent links. The corresponding STD and TDEV are further used to characterize the statistical dispersion and temporal stability of the residual difference. Considering the independently calibrated optical-fibre reference system, the results demonstrate close sub-nanosecond agreement between the BDS RTPPP and optical-fibre time transfer solutions.

**Figure 9:**
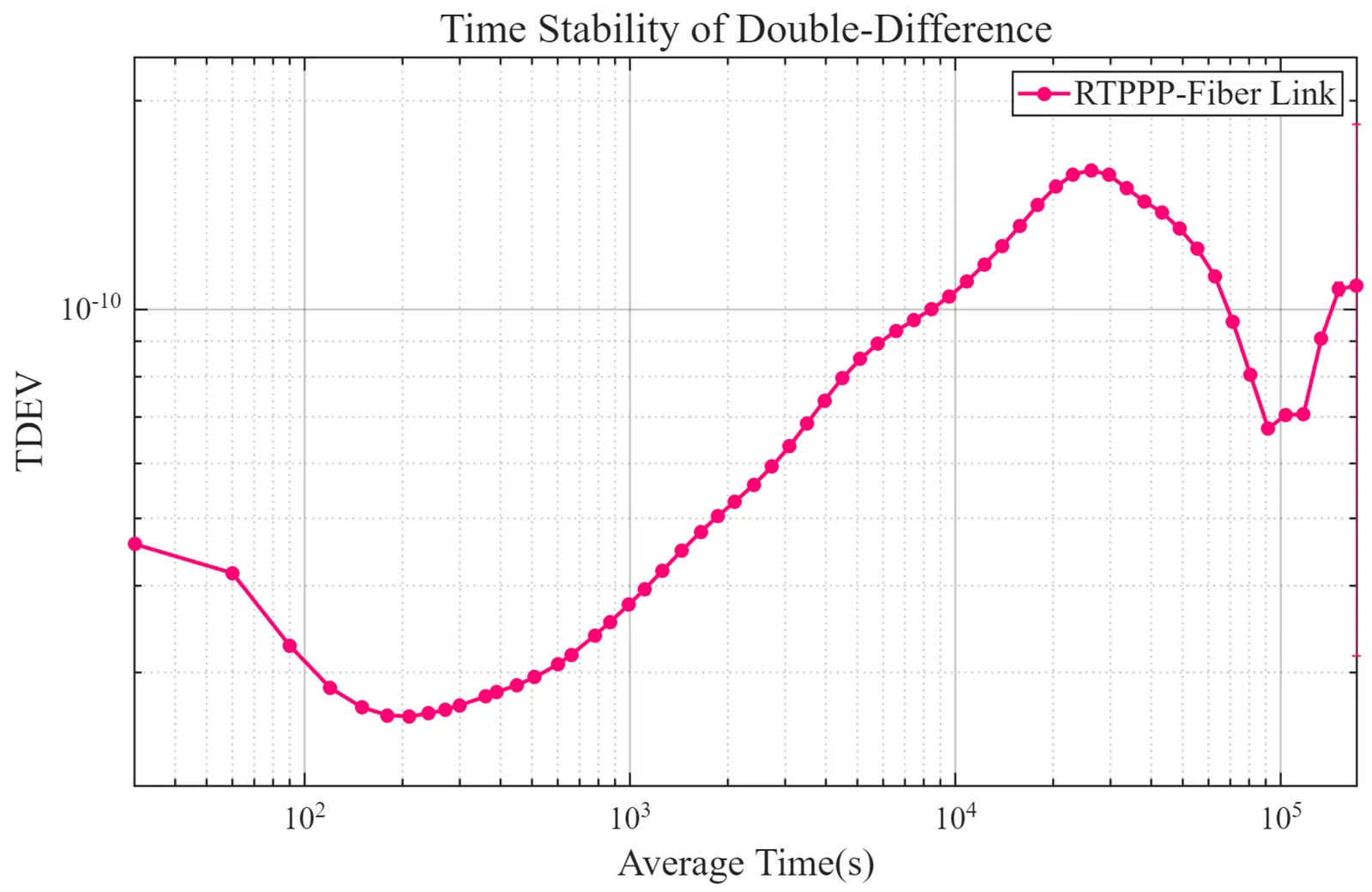
TDEV of the residuals difference that RTPPP results relative to optical-fibre link.

Based on the difference between BDS RTPPP and optical-fibre link, TDEV was further calculated to characterize the temporal stability of the link residuals, as shown in Figure 9. The TDEV values are 4.59 × $10^{-11}$ and 4.11 × $10^{-11}$ at averaging time of 30 s and 1200 s, respectively, and remain at approximately 7.07 × $10^{-11}$ at 86,400 s. Overall, the differential results maintain good temporal stability over the investigated averaging intervals. It should be emphasized that TDEV is used here to characterize the time stability of the difference between the BDS RTPPP and optical-fibre results, and serves as an estimate of the corresponding statistical uncertainty contribution of the comparison, consistent with the statistical evaluation framework described in Section 2.4.

**Figure 10:**
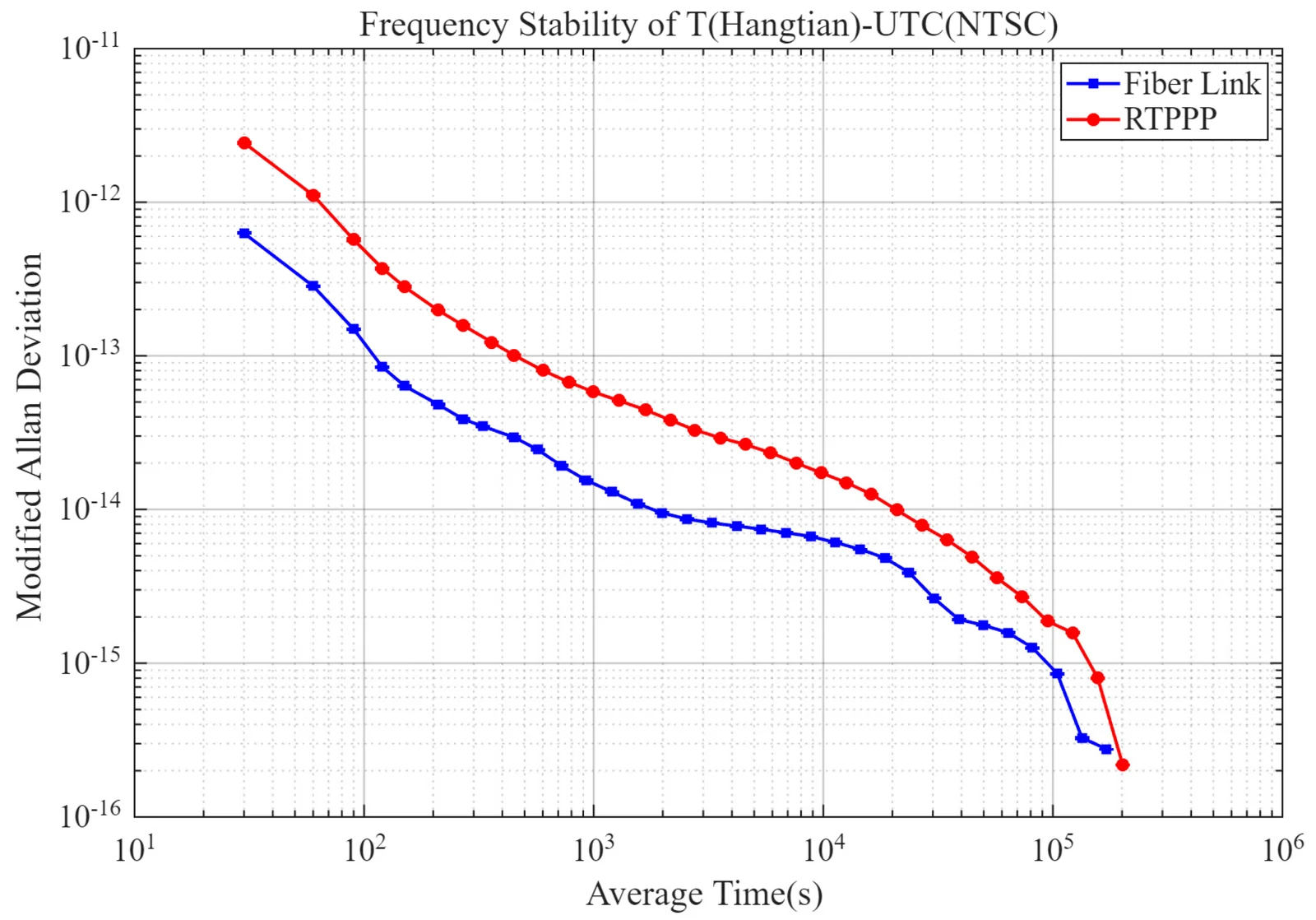
Frequency stability of T(Hangtian) − UTC(NTSC) obtained from the BDS RTPPP and optical-fibre links.

Figure 10 shows the frequency stability of T(Hangtian) − UTC(NTSC) obtained from the BDS RTPPP and optical-fibre links. At the averaging time of 30 s and 1200 s, the MDEV values of BDS RTPPP are 2.44 × $10^{-12}$ and 5.27 × $10^{-14}$, respectively, compared with 6.28 × $10^{-13}$ and 1.31 × $10^{-14}$ for the optical-fibre link. At an averaging time of 86,400 s, the frequency stability reaches approximately 2.07 × $10^{-15}$ for BDS RTPPP and 1.19 × $10^{-15}$ for the optical-fibre. It clearly indicates that optical-fibre link exhibit better frequency stability over most averaging time. Nevertheless, the MDEV of the BDS RTPPP results decreases continuously with increasing averaging time and reaches the $10^{-15}$ level at one day, demonstrating its capability to characterize the long-term frequency behaviour of T(Hangtian).

**Figure 11:**
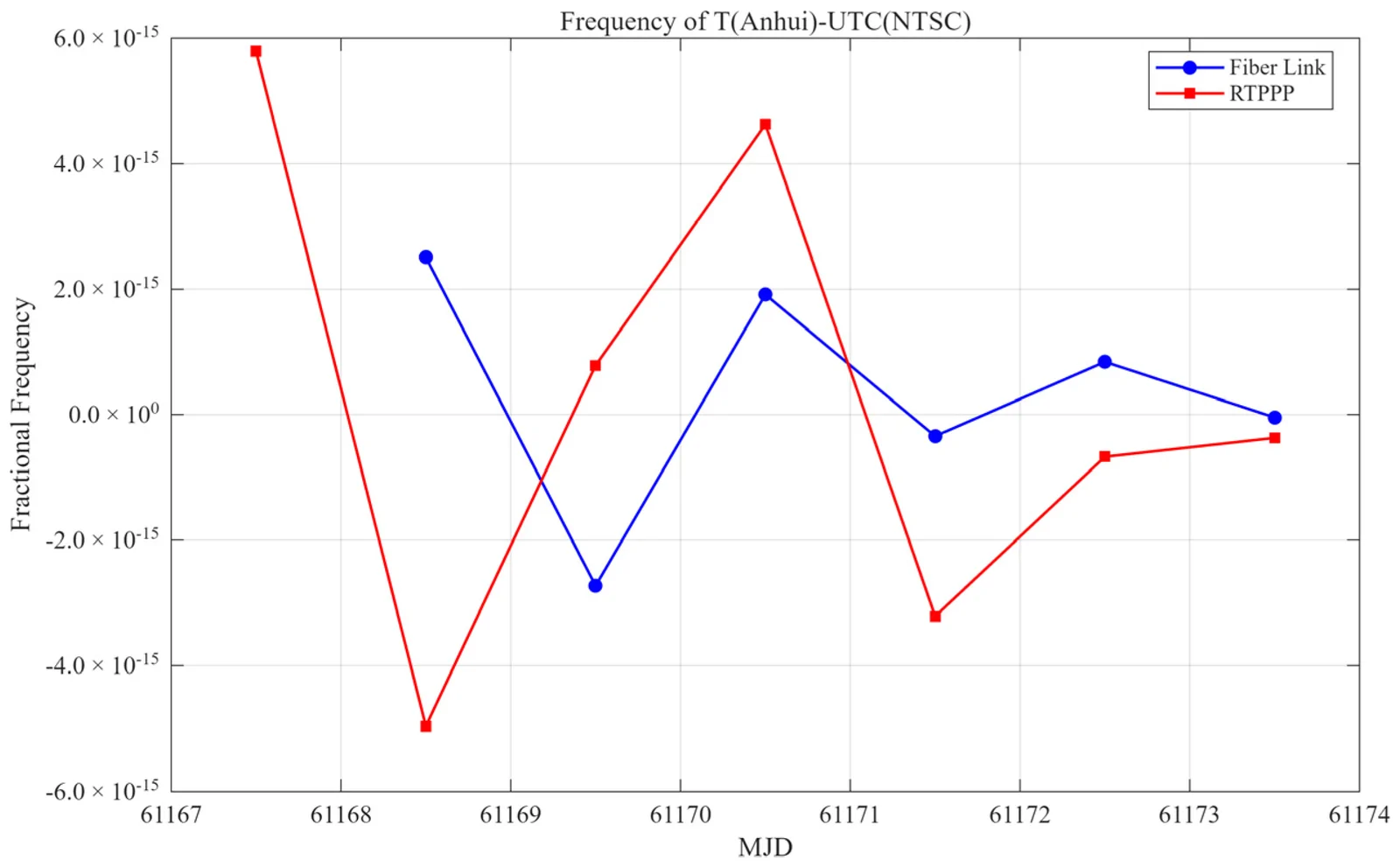
Daily frequency deviations of T(Hangtian) and UTC(NTSC) between RTPPP and Optical Fibre.

Figure 11 shows the daily relative frequency deviations of T(Hangtian) with respect to UTC(NTSC) obtained from the BDS RTPPP and optical-fibre links. The two solutions exhibit generally consistent temporal variations, although the BDS RTPPP results show somewhat larger day-to-day fluctuations. Because the optical-fibre observations at the beginning of the experiment do not cover a complete 1-day interval, direct daily frequency comparisons were performed over the five complete common intervals from MJD 61169 to MJD 61174. To directly quantify the agreement between the two links, the daily relative frequency differences and residual frequency differences between BDS RTPPP and optical-fibre link were calculated, as summarized in Table 3. The mean absolute relative frequency deviations obtained from BDS RTPPP and the optical-fibre link are 1.93 × $10^{-15}$ and 1.18 × $10^{-15}$, respectively. More importantly, the mean residual frequency difference between the two links is 2.80 × $10^{-16}$, while the daily mean absolute residual frequency difference is 2.21 × $10^{-15}$. These results indicate good systematic agreement between the BDS RTPPP solution and the independently calibrated optical-fibre reference. The STD of the residual frequency differences is 2.74 × $10^{-15}$, which reflects the day-to-day statistical variation in the two link comparisons rather than the systematic metrological calibration contribution.

### 4.3. Long-Baseline Comparison Experiments

**Figure 12:**
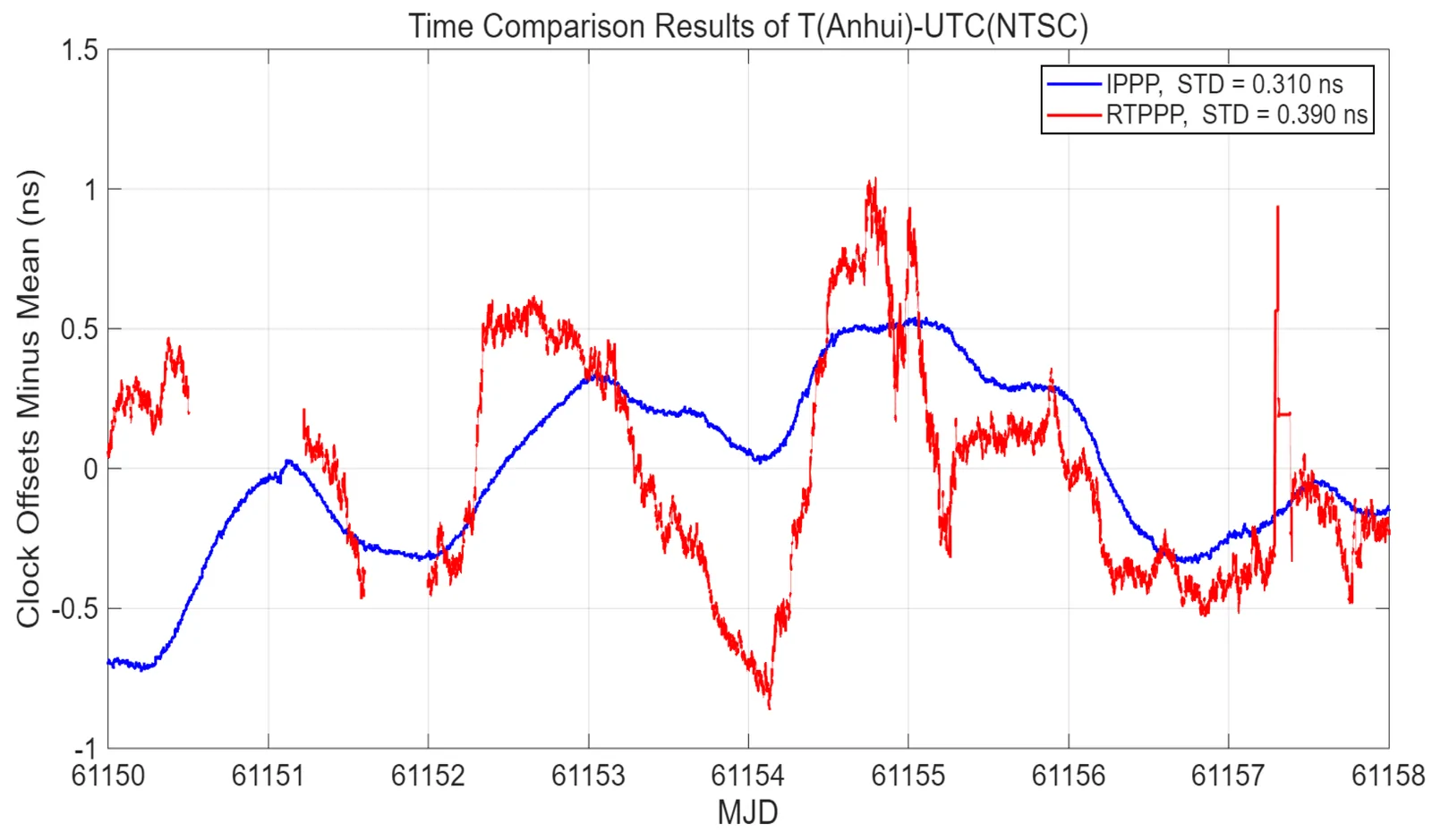
T(Anhui) − UTC(NTSC) obtained from BDS RTPPP and IPPP.

Using the long-baseline configuration described in Section 3, the BDS RTPPP results for T(Anhui) were evaluated against the independent multi-GNSS IPPP reference. After link-delay compensation, the T(Anhui) − UTC(NTSC) results obtained from the two links are presented in Figure 12.

After link delay compensation, the T(Anhui) − UTC(NTSC) results obtained from BDS RTPPP and IPPP are presented in Figure 12, where the red curve denotes the BDS RTPPP solution and the blue curve denotes the IPPP solution. The two-time transfer results exhibit generally consistent temporal variations, while the BDS RTPPP results show somewhat larger short-term fluctuations. The STDs of the BDS RTPPP and IPPP time transfer results are 0.39 ns and 0.31 ns, respectively. However, these values characterize the overall fluctuations of the measured T(Anhui) − UTC(NTSC) and contain contributions from both the intrinsic behaviour of the remote timekeeping system and the corresponding time transfer links. Therefore, the individual STDs are not used here as direct indicators of link accuracy or time transfer performance. Instead, the differences between RTPPP and IPPP are calculated to evaluate the agreement between the two links, while TDEV and MDEV are used to characterize their time and frequency stability, respectively.

**Figure 13:**
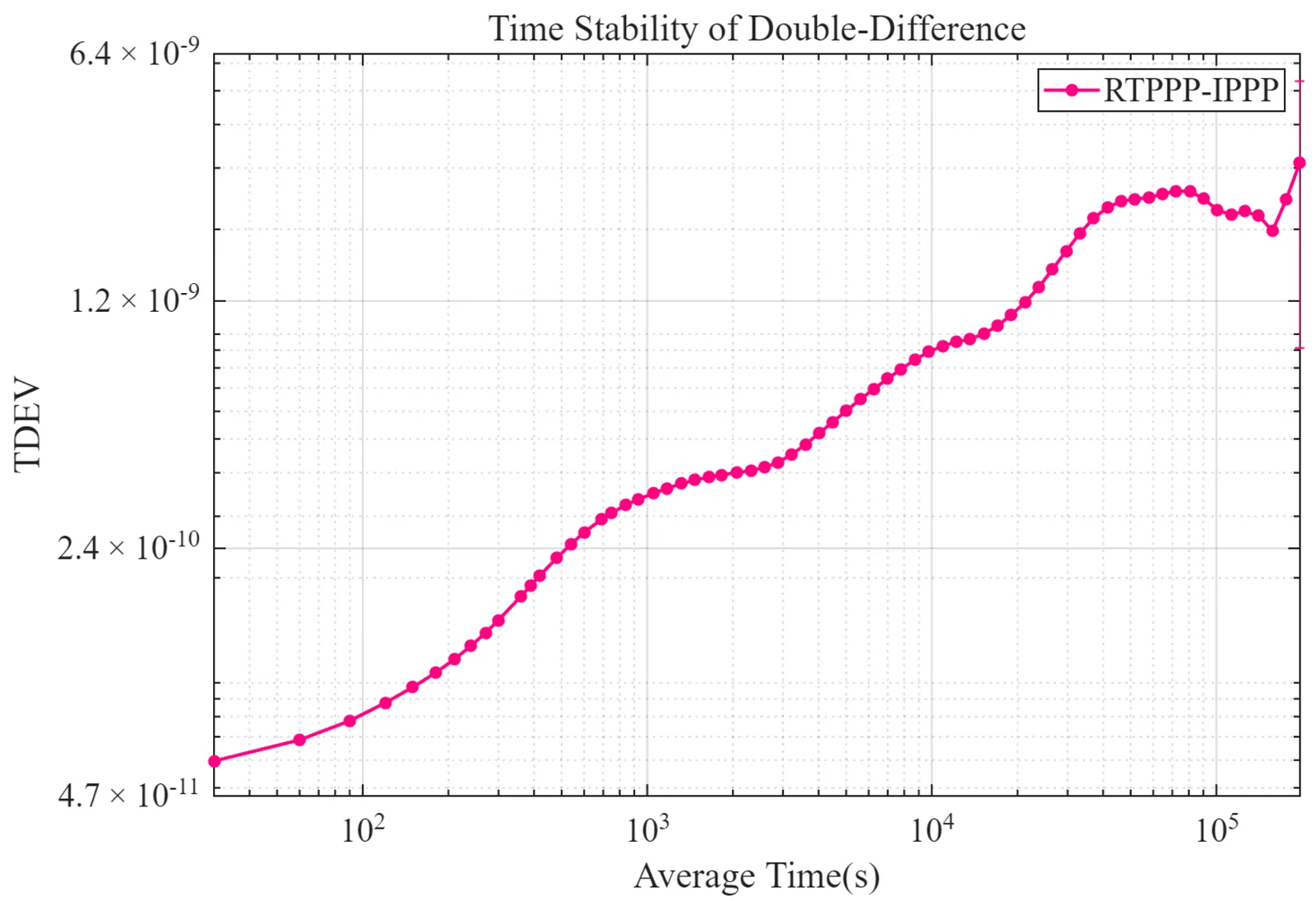
TDEV of the link-difference results between BDS RTPPP and the IPPP.

The differences between RTPPP and IPPP were further utilized to remove the common variation in T(Anhui) − UTC(NTSC), and its TDEV is shown in Figure 13. Compared with the individual STDs of the two links, the differential results provide a more appropriate basis for evaluating the link agreement because the common variation in the remote timekeeping system is largely suppressed. TDEV is used here to characterize the temporal stability of the link residuals rather than the absolute metrological calibration accuracy.

The difference series between BDS RTPPP and IPPP has an STD of 0.08 ns. The corresponding TDEV values are 5.97 × $10^{-11}$ s at 30 s and 3.63 × $10^{-11}$ s at 1200 s, and reach 2.22 × $10^{-9}$ s at 120,000 s. Overall, the TDEV remains within the $10^{-11}$–$10^{-9}$ s range over the investigated averaging intervals. According to the statistical uncertainty evaluation framework described in Section 2.4, these TDEV values characterize the time stability of the link difference and are used to estimate the corresponding statistical uncertainty contribution of the BDS RTPPP comparison.

**Figure 14:**
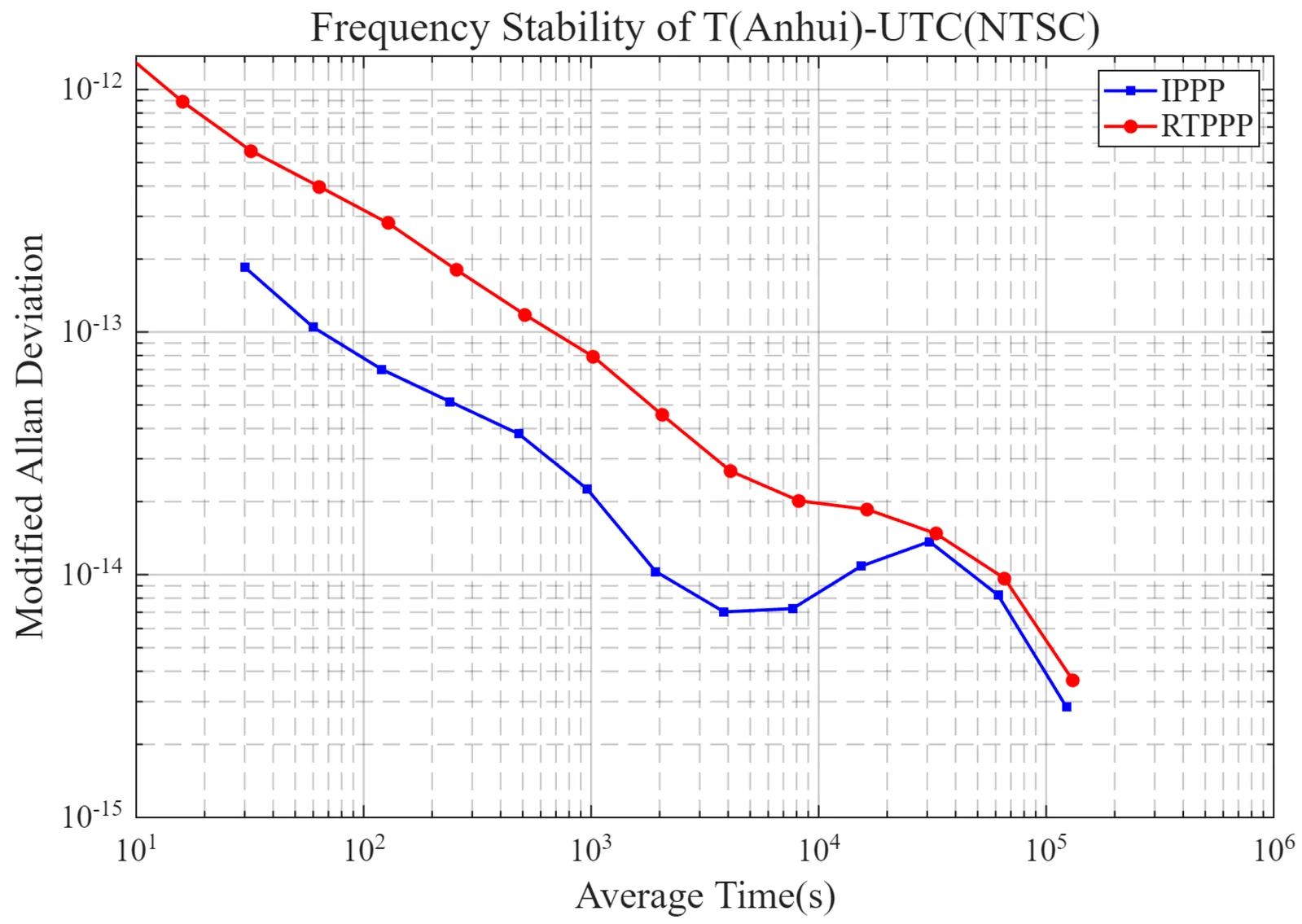
Frequency stability of T(Anhui) − UTC(NTSC) derived from the BDS RTPPP and IPPP.

Figure 14 shows the MDEV results obtained from the BDS RTPPP and IPPP links. At the averaging time of 30 s and 1200 s, the MDEV values of BDS RTPPP are 6.00 × $10^{-13}$ and 7.33 × $10^{-14}$, respectively, compared with 1.85 × $10^{-13}$ and 1.95 × $10^{-14}$ for IPPP. At an averaging time of 86,400 s, the frequency stability reaches 7.95 × $10^{-15}$ for BDS RTPPP and 6.12 × $10^{-15}$ for IPPP. It clearly demonstrated that the frequency stability of IPPP superior to BDS RTPPP over the most averaging time, whereas the BDS RTPPP solution also reaches the $10^{-15}$ level at one day.

**Figure 15:**
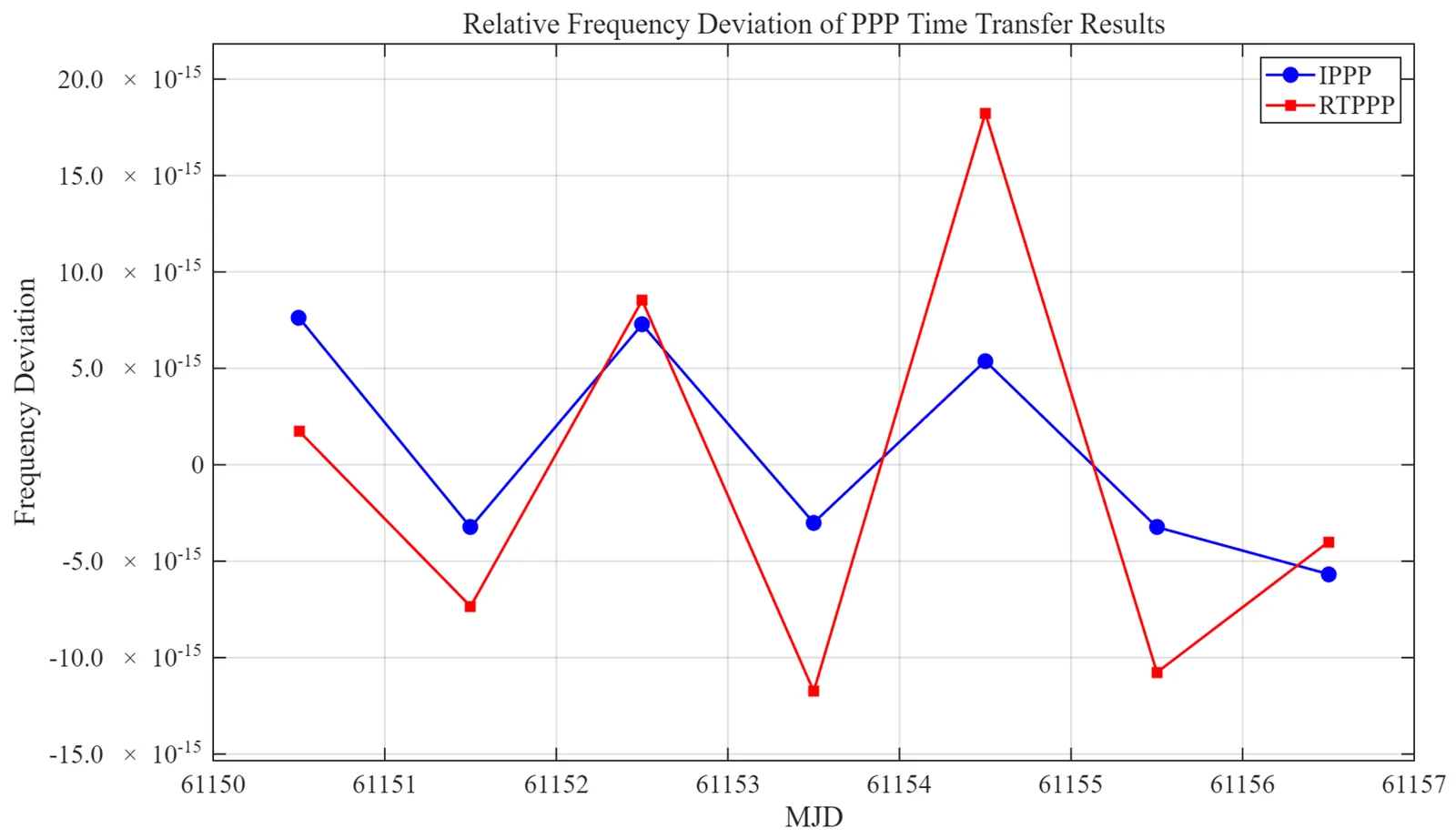
Comparison of relative frequency deviations between T(Anhui) and UTC(NTSC).

Figure 15 shows the daily relative frequency deviations of T(Anhui) with respect to UTC(NTSC) obtained from the BDS RTPPP and IPPP links. The BDS RTPPP results exhibit larger fluctuations than the IPPP results, while the overall temporal variations in the two solutions remain broadly consistent. To directly quantify the agreement between the two links, the residual frequency difference between BDS RTPPP and IPPP were further calculated, as summarized in Table 4. The mean absolute relative frequency deviations obtained from BDS RTPPP and IPPP are 8.90 × $10^{-15}$ and 5.06 × $10^{-15}$, respectively. More importantly, the absolute mean residual frequency difference between BDS RTPPP and IPPP is 1.49 × $10^{-15}$, while the daily mean absolute residual frequency difference is 5.94 × $10^{-15}$. These results provide a more direct assessment of the systematic agreement between the BDS RTPPP and IPPP solutions than the stability metrics alone. The STD of the residual frequency difference is 7.42 × $10^{-15}$, reflecting the statistical variation in the link difference.

## 5. Conclusions and Outlook

In this study, a remote real-time performance evaluation method for high-precision timekeeping systems based on BDS RTPPP is developed. A second-order receiver clock model is adopted in the RTPPP processing to provide temporal constraints on station-clock estimation. Previous studies have demonstrated the benefits of such receiver clock modelling for improving PPP-B2b solution continuity and robustness [23]. In the present work, the primary contribution is the establishment and experimental validation of a complete remote performance evaluation framework based on BDS RTPPP, including common-clock link-delay calibration and independent short- and long-baseline reference comparisons.

Short-baseline experiments show that the STD of BDS RTPPP time transfer is 0.186 ns, demonstrating sub-nanosecond level time-transfer performance. For T(Hangtian), the frequency stability at the averaging time of one day obtained from BDS RTPPP is 2.07 × $10^{-15}$. Based on the complete common daily intervals, the mean absolute relative frequency deviation obtained from BDS RTPPP is 1.93 × $10^{-15}$. Moreover, the mean residual daily frequency difference between BDS RTPPP and the optical-fibre reference is 2.80 × $10^{-16}$, and the mean absolute daily frequency difference is 2.21 × $10^{-15}$. These metrics closely agree with optical-fibre benchmarks, verifying that BDS RTPPP can faithfully characterize the actual performance of T(Hangtian). For the long-baseline experiment, the BDS RTPPP time transfer results for T(Anhui) − UTC(NTSC) has an STD of 0.39 ns; which characterizes the overall fluctuation of the measured time offset series rather than the absolute metrological calibration uncertainty. The BDS RTPPP frequency stability reaches 7.95 × $10^{-15}$ at an averaging time of one day, while the mean absolute relative frequency deviation over the observation period is approximately 8.90 × $10^{-15}$. The mean residual daily frequency difference between BDS RTPPP and IPPP is −1.49 × $10^{-15}$, corresponding to an absolute mean residual of 1.49 × $10^{-15}$, and the mean absolute daily frequency difference is 5.94 × $10^{-15}$. The frequency stability measured from RTPPP aligns well with IPPP solutions, enabling accurate depiction of the actual performance of T(Anhui).

Although the performance evaluation results of the short-baseline and long-baseline RTPPP links are subject to error sources including cable propagation delays, PLL discrepancies between the receiver’s internal and external frequency references, precise orbit/clock products, real-time corrections, and customized data processing strategies, the proposed BDS RTPPP approach enables real-time, high-precision, online remote continuous monitoring and evaluation for distributed timekeeping systems. Benefiting from its compact hardware configuration and flexible on-site deployment while maintaining outstanding evaluation precision, this method can be widely generalized to scenarios covering remote metrology institutions, multi-mode collaborative timekeeping networks and municipal-grade time reference systems.

Future work will strengthen RTPPP link robustness and develop combined multi-technique schemes combining GNSS, optical-fibre, and other time transfer techniques to improve system reliability in complex environments. For remote and communication-restricted evaluation scenarios, supplementary communication approaches including the BeiDou short-message service, digital broadcasting, and LEO satellites links will also be investigated.

## Figures and Tables

**Table 1:** Geodetic Stations and Associated Equipment for Selected timekeeping Laboratories.

Station	Time Link	Equipment Mode	Antenna	External Reference
Lintong	BDS RTPPP	NTSC-PPP	ARF-AS1-3D-FS_RF-TECH	UTC(NTSC)
	GNSS IPPP	SeptentrioPolaRx5TR	SEPCHOKE_MC	
	Optical-Fibre	/	/	
Hangtian	BDS RTPPP	NTSC-PPP	ARF-AS1-3D-FS_RF-TECH	T(Hangtian)
	Optical-Fibre	/	/	
Anhui	BDS RTPPP	NTSC-PPP	ARF-AS1-3D-FS_RF-TECH	T(Anhui)
	GNSS IPPP	SeptentrioPolaRx5TR	SEPCHOKE_MC	

**Table 2:** IPPP parameter estimation and error correction strategies.

Error/Delay	Correction Strategy
Precise products	CODE precise orbit, clock, and OSB bias products
Parameter estimation	Extended Kalman Filter
Ionospheric delay	GPS + Galileo dual-frequency ionosphere-free combination
Tropospheric delay	The dry component is corrected using the Saastamoinen model, and the wet component is mapped by GMF and estimated as a ZTD parameter
Ambiguity fixing	Wide-lane satellite single-difference ambiguities are fixed by the MW combination, and narrow-lane ambiguities are resolved using the LAMBDA algorithm
Phase wind-up	Corrected using the Wu model
Tidal correction	Corrected following the IERS 2010 model
Antenna phase center	Antenna PCO/PCV corrections adopt the IGS24.atx file

**Table 3:** Daily relative frequency deviations and link residual differences between BDS RTPPP and the optical-fibre reference.

MJD	Optical-Fibre	RTPPP	RTPPP − Optical-Fibre
61169	−2.73 × $10^{-15}$	7.85 × $10^{-16}$	3.51 × $10^{-15}$
61170	1.92 × $10^{-15}$	4.63 × $10^{-15}$	2.71 × $10^{-15}$
61171	−3.45 × $10^{-16}$	−3.22 × $10^{-15}$	−2.87 × $10^{-15}$
61172	8.42 × $10^{-16}$	−6.72 × $10^{-16}$	−1.51 × $10^{-15}$
61173	7.35 × $10^{-17}$	−3.71 × $10^{-16}$	−4.44 × $10^{-16}$

**Table 4:** Daily relative frequency deviations and residual frequency differences between BDS RTPPP and IPPP.

MJD	IPPP	RTPPP	RTPPP − IPPP
61150–61151	7.63 × $10^{-15}$	1.82 × $10^{-15}$	−5.80 × $10^{-15}$
61151–61152	−3.24 × $10^{-15}$	−7.43 × $10^{-15}$	−4.19 × $10^{-15}$
61152–61153	7.27 × $10^{-15}$	8.65 × $10^{-15}$	1.38 × $10^{-15}$
61153–61154	−3.00 × $10^{-15}$	−1.18 × $10^{-14}$	−8.77 × $10^{-15}$
61154–61155	5.38 × $10^{-15}$	1.81 × $10^{-14}$	1.27 × $10^{-14}$
61155–61156	−3.23 × $10^{-15}$	−1.05 × $10^{-14}$	−7.25 × $10^{-15}$
61156–61157	−5.67 × $10^{-15}$	−4.12 × $10^{-15}$	1.55 × $10^{-15}$

## Data Availability

The raw data supporting the conclusions of this article will be made available by the authors on request.

## Abbreviations

The following abbreviations are used in this manuscript:

ACC	Analysis Center Coordinator
AV	ALL-in-View
BDS	BeiDou Navigation Satellite System
CNES	Centre National d’Études Spatiales
CV	Common-View
EKF	Extended Kalman Filter
RTPPP	Real-Time Precise Point Positioning
PPP-B2b	Precise Point Positioning B2b service provided by BDS
IPPP	Precise Point Positioning of Integer Ambiguity
PPP-AR	Precise Point Positioning with Ambiguity Resolution
GNSS	Global Navigation Satellite System
GPS	Global Positioning System
UTC(NTSC)	Coordinated Universal Time of the National Time Service Center
NTSC	National Time Service Center, Chinese Academy of Sciences
IGS	International GNSS Service
ISB	Inter-System Bias
CODE	Center for Orbit Determination in Europe
MGEX	Multiply GNSS Experiments
MJD	Modified Julian Date
SSR	State Space Representation
STD	Standard Deviation
MDEV	Modified Allan Deviation
TDEV	Time Deviation
OSB	Observable-Specific Bias
OCXO	Oven-Controlled Crystal Oscillator
PCO	Phase Center Offset
PCV	Phase Center Variation
